# Association of SNPs in exon 2 of the MHC B-F gene with immune traits in two distinct chicken populations: Chinese Beijing-You and White Leghorn

**DOI:** 10.1080/09064700902988905

**Published:** 2009-05-29

**Authors:** L. B. Liu, C. M. Wu, J. Wen, J. L. Chen, M. Q. Zheng, G. P. Zhao

**Affiliations:** Institute of Animal Science, Chinese Academy of Agricultural Science, Beijing 100094, China

**Keywords:** Chicken, major histocompatibility complex (MHC), B-F gene, immune traits, single nucleotide polymorphism (SNP)

## Abstract

Antibody titers raised for vaccinations against avian influenza (AI) and Newcastle disease (ND) were higher in Chinese Beijing-You (BJY) than in White Leghorn (WL) (*P* < 0.001), but there was no breed difference in titers for sheep red blood cells (SRBC). Genotyping by PCR-SSCP identified seven haplotypes in WL and 17 in BJY. After sequencing PCR products (35 and 85, respectively), 43 (WL) and 47 (BJY) single nucleotide polymorphisms (SNPs) were found in the 264 bp of exon 2. In WL chickens, significant associations were found with antibody responses to AI (two SNPs), ND (six SNPs), and SRBC (one SNP), while in BJY there was association with responses to ND (two SNPs) and SRBC (two SNPs), but none with AI. These results indicate that the genomic region bearing exon 2 of the major histocompatibility complex B-F gene has significant effects on antibody responses to SRBC and vaccination against AI and ND. Different SNPs affected antibody titers for each of the antigens and they differed between these very distinct breeds.

## Introduction

The major histocompatibility complex (MHC) molecules play important roles in the regulation of the immune response by communicating among different cellular components of the immune system: T cells, B cells, and antigen-presenting cells (Lamont, 1998a,b,c). The MHC in chickens is known to have a significant association to disease resistance and susceptibility to numerous pathogens including Marek's disease virus (Bacon et al., 2001), Rous sarcoma tumor virus (Taylor, 2004), and Salmonella (Cotter et al., 1998). The association between MHC and disease outcome is found in laboratory strains of chickens and commercially important flocks (Hansen et al., 1967; Bacon et al., 1981). The crucial function of the MHC in the immune response therefore makes it a promising candidate region for genetic selection to improve avian disease resistance.

The chicken MHC is located on chromosome 16 (Jacob et al., 2000) and contains three classes of MHC molecules identified by alloantisera: B-F (Class I), B-L (Class II), and B-G antigen (Kaufman & Lamont, 1996). The Class I molecules (encoded by the B-F gene) contain an α chain and non-covalently associated β2 m chain. The α chain genes are composed of the signal region, extracellular domains (α1,α2,α3), transmembrane region, cytoplasmic tail, and a peptide-binding cleft formed by folded α1 and α2 domains (Kaufman & Lamont, 1996). Only one Class I molecule in the chicken MHC is highly expressed, leading to strong genetic associations with infectious pathogens (Koch et al., 2007). However, Class I molecule's variability occur in exons 2 and 3, encoding the α1 and α2 domains chiefly and exon 2 is more highly polymorphic (Livant et al., 2004).

Chickens are vulnerable to a large variety of pathogens, including important zoonotic organisms such as avian influenza (AI) H5N1. Among the potential immune response loci, the chicken MHC can determine striking resistance or susceptibility to infectious pathogens, as well as response to vaccines (Bacon, 1987; Bacon et al., 1987; Plachy et al., 1992). Many studies have demonstrated associations between the chicken MHC and immune traits, particularly antibody production against a variety of antigens such as: *Brucella abortus*, Newcastle disease virus (NDV) and sheep red blood cells (SRBC) (Dunnington et al., 1992), and antibody titer to infectious bursal disease virus (Ewald et al., 2007). Liu et al. (2008) demonstrated that the Cytotoxic T Lymphayte (CTL) response of chickens exposed to infectious bronchitis virus could be evaluated effectively using the prepared MHC-I BF2*15/peptide tetramer.

Most of the research on MHC BF2 genes in chickens has used White Leghorns (WL) as an experimental animal (Ewald & Livant, 2004) and very little is known about the association between MHC and disease resistance in indigenous chicken breeds that are known generally to have superior genetic resistance to disease. Beijing-You (BJY) is a famous Chinese native chicken with good meat taste, but has an unimpressive growth rate and feed conversion rate compared to the conventional broilers. Leghorns and BJY have very different genetic backgrounds which were exploited here in seeking single nucleotide polymorphisms (SNPs), which might be used as markers for improved immune function and aid in illustrating promiscuous peptide binding of MHC Class I. The objectives of this study were to examine and compare diversity of exon 2 of the MHC B-F gene and its association with immune traits in these two distinct chicken populations.

## Materials and methods

### Experimental animals and vaccination procedure

Chickens (WL and BJY, 250 of each breed) were from stocks maintained at the Institute of Animal Science at the Chinese Academy of Agricultural Sciences (CAAS). BJY and WLs used in this study were two outbred populations which were maintained as conserved resource at CAAS for more than 15 years. There are more than 300 females and 60 males that were randomly chosen to produce progeny in each generation for the two breeds, respectively. Peripheral blood was collected from all the chickens on days 50, 71, 100, and 134. Part of each sample from day 50 was collected using 0.5% Ethylene Diamine Tetraacetic Acid (EDTA) as anticoagulant (for DNA isolation) and the remainder was allowed to clot. Serum was obtained from all samples after centrifuging at 3000 × *g* for 5 min. The standard vaccination protocol for all birds is described in Table I. Birds were challenged with SRBC at day 128.

**Table I:** Immunization protocol.

Age/d	Vaccine	Vaccination route	Remarks
0	Marek's disease vaccine	Subcutaneous injection	Marek's disease cryophilic vaccine (CV1988/Rispens)
7	Newcastle disease (Lasota strain), Infectious bronchitis (H120 strain) virus vaccine	Eye-drop and nose-drop	Live freeze-dried vaccine
15	Avian Influenza (AI) and Newcastle disease (ND) virus oil adjuvant vaccine	Subcutaneous injection	Recombinant inactivated vaccine (H5N1 Subtype, Re-4 strain), ND inactivated vaccine
28	Avian Influenza (AI) and Newcastle disease (ND) virus oil adjuvant vaccine	Subcutaneous injection	Similar to 15 d of age

### Immunological assays

Serum titers of antibodies against AI and Newcastle disease (ND) viruses were determined by inhibition of agglutination. Serial dilutions (1:2 to 1:2048) of serum were made in 96-well, V-bottom micro titer plates containing 50 μl of Phosphate Buffered Saline (PBS) in all wells. Antigen (50 μl of NDV or Avian Influenza Virus (AIV), four hemagglutination units, Harbin Veterinary Research Institute, CAAS, China) was added to all of the wells except the last row, which served as controls. The Ag-serum mixture was pre-incubated for 10 min at 37°C, then a suspension of 1% Specific Pathogen Free (SPF) rooster erythrocytes (50 μl) was added and incubated for 30 min. Appropriate controls were included. The highest dilution of serum (expressed as reciprocal log_2_ values) causing complete inhibition was considered to be the titer.

### Antibody response to challenge with sheep red blood cells (SRBC)

The birds were injected with 1 ml of 25% SRBC (pooled from 5 to 7 unrelated sheep) diluted in PBS at 128 d of age, 6 d before the blood sampling on day 134. Antibody titer was determined by the method described by Hudson and Hay (1976).

### Genomic sequencing of the major histocompatibility complex (MHC) B-F exon 2

Genomic DNA was extracted from day 50 erythrocytes using the standard procedure (Sambrook et al., 1989). All primers used for amplification of exon 2 (encoding α1 domains) of B-F genes were designed using the Oligo 6.0 program and were based on the chicken B-F sequence (GenBank accession no. M31012); they were obtained from Sangon Co. Ltd, Shanghai. The expected size of the product, including the entire exon 2, was 396 bp. The upstream primer (5′-CGCCCGTAACCCCACCC-3′) was taken from intron 1, and the downstream primer (5′-AGCCCATCCCACACCCACGG-3′) was from intron 2. The PCR reaction mix consisted of 2.5 μl of 10 × buffer, 0.2 mM of each dNTP, 1 μl of each primer, 1 μl of genomic DNA, and 0.5 U of DNA polymerase (TaKaRa Biotechnology Co. Ltd., Dalian, China) in a final volume of 25 μl. The reaction conditions consisted of an initial 10 min hold at 95°C, 35 cycles of 95°C for 1 min, 62°C for 2 min and 72°C for 1 min, and a final 10 min extension step at 72°C. The quantity and integrity of the products were examined after electrophoresis on 2% agarose gel and staining with 0.25 μg/ml ethidium bromide.

### Polymerase Chain Reaction–Single Strand Confirmation Polymorphism (PCR-SSCP) analysis

Three microliters of PCR reaction product were denatured at 95°C in 7 μl formamide/dye for 10 min and immediately chilled on ice to prevent reannealing. Denatured PCR products were subject to SSCP analysis in 8% polyacrylamide gels at 150 V and 4°C for 22 h in 0.5 × TBE buffer (Oto et al., 1993). The bands were visualized after staining with silver.

### Major histocompatibility complex (MHC) B-F exon 2 sequencing

Exon 2 sequencing was performed in both directions with the upstream and downstream primers described above. After eliminating flanking intronic portions to obtain just exon 2, sequences were compared using the DNASRAT6.0 software package.

### Statistical analyses

All the antibody titers of SRBC, AI, and ND were presented as log transformed value and the arithmetic means of these log values were calculated. The difference of antibody titer between breeds and the association between SNP alleles and overall immune traits were analyzed with ANOVA program of SAS software version 8.02 (SAS Institute, 1999). The arithmetic mean of AI and ND titer across all sampling days (50, 71, 100, and 134) was calculated for each bird and used in the analysis of association.

## Results

### Comparison of avian influenza (AI), Newcastle disease (ND), and sheep red blood cells (SRBC) titers between the two breeds

There were significant differences between the two breeds in AI titers, with those in BJY exceeding those of WL (*P* < 0.001) at all sampling days (Table II); highest titers in both breeds were at the 100th d. Titers against NDV were higher in BJY than WL at 50 (*P* < 0.001), 100 (*P* < 0.05), and 134 d (*P* < 0.001), and not different at the 71st d; and highest titers were measured on 50th (BJY) and 71st d (WL). There was no significant difference of SRBC titers between two breeds.

**Table II:** Antibody titers of AI, ND, and SRBC in two breeds (log_2_)^1^.

		Antibody titers			
		
Antibody	Breed	50 d	71 d	100 d	134 d
AI	WL	8.55±1.59^B^	9.29±1.31^B^	9.99±1.14^B^	3.48±0.96^B^
	BJY	9.26±1.12^A^	9.69±1.16^A^	10.34±1.09^A^	4.45±1.52^A^
ND	WL	10.36±0.96^B^	10.51±0.82	9.30±1.40^b^	7.81±1.38^B^
	BJY	10.78±0.58^A^	10.55±1.08	9.45±1.51^a^	9.00±1.13^A^
SRBC	WL				8.64±1.54
	BJY				8.61±1.18

1 Means (±SD) are each based upon 193–250 individuals. Within a column, values followed by different uppercase letters are significantly different (*P* < 0.01) and values followed by different lowercase letters are significantly different (*P* < 0.05).

### Comparison between breeds of DNA and protein consensus sequences

Different bands revealed by PCR-SSCP of the MHC B-F exon 2 indicated the minimal number of allelic forms of this region of the gene and each was identified as being a distinct haplotype. Using 250 chickens from each breed, seven haplotypes were found in WL and 17 haplotypes in BJY. Five of PCR products within each haplotype (35 WL and 85 BJY) were sequenced. The exon 2 sequences (264 bp, α1 domain) were highly homologous, but 43 SNPs were found in WL and 47 SNPs in the BJY. The SNPs were exclusively base substituted (there were no insertions or deletions) and resulted in a number of AA changes, as detailed in Table III. Alignment of the 120 (35 + 85) DNA sequences showed that 35 out of the 55 positions occurred in both WL and BJY breeds; eight SNP positions in the WL and 12 SNP positions in the BJY were breed specific.

**Table III:** SNPs from exon 2 of MHC B-F haplotypes in White Leghorn (WL) and Beijing-You (BJY).

Location^a^ and SNP	AA and change	Breed
9 A/T	4 T/S	WL
21 A/G	8 I/V	WL
24 T/C	9 Y/H Y/S	WL/BJY
25 C/A/G	9 Y/H Y/S	WL/BJY
26 A/T	9 Y/H Y/S Y/R	BJY
55 T/A	19 Q/L	WL/BJY
64 T/A	22 F/Y	WL/BJY
69 A/G	24 T/A T/I T/D T/N	WL/BJY
70 C/T/A	24 T/I T/D T/N T/A	BJY
71 T/C	24 T/I T/D T/N	BJY
80 C/T	27 Y/Y	WL/BJY
99 A/G	34 V/M	WL/BJY
100 T/C	34 V/A	WL
119 G/T	40 A/A	WL/BJY
126 G/T	43 V/Y V/D	WL/BJY
127 A/C	43 V/Y V/D	WL/BJY
128 C/T	43 V/Y V/D Y/D	WL/BJY
149 G/A	50 I/M	WL/BJY
158 G/C	53 K/N	WL/BJY
159 A/G	54 A/T	WL/BJY
177 G/A	60 D/N	BJY
179 T/C	60 D/D D/D N/N	WL/BJY
180 G/A	61 G/RG/S G/R	WL/BJY
182 A/T	61 G/RG/S	WL
183 C/G	62 Q/E	WL/BJY
192 A/C	65 I/LI/M	BJY
193 T/C	65 I/T	WL
194 G/C	65 I/LI/M	BJY
195 G/T	66 G/V	WL
196 G/T	66 G/V	WL/BJY
197 A/C	66 G/GV/V	BJY
201 G/C	68 G/R	WL/BJY
204 A/C	69 N/H	BJY
211 A/G	71 Q/R	WL/BJY
213 A/G	72 I/V I/A I/S I/T	BJY
214 T/G/C	72 I/S/T	WL/BJY
216 G/A	73 D/N	BJY
217 A/T	73 D/V	WL/BJY
218 C/G	73 D/N D/V	WL/BJY
219 C/A	74 R/S R/N R/K	WL/BJY
220 G/A	74 R/S R/N R/K	WL/BJY
221 C/A	74 R/S R/N R/K	WL/BJY
223 A/T	75 E/V D/V	WL/BJY
224 G/T	75 E/V D/V	WL/BJY
226 A/G	76 N/S	WL/BJY
228 C/T	77 L/L	WL
231 G/A	78 G/N G/S	WL
232 A/G	78 G/D N/S G/D	WL/BJY
235 T/C	79 I/T	WL/BJY
238 T/G	80 L/R	BJY
241 G/A	81 Q/R	BJY
243 C/G	82 R/GR/E	WL/BJY
244 G/A	82 R/Q R/E	WL/BJY
245 G/A	82 R/QR/EE/G	WL/BJY
248 C/A	83 R/R	WL/BJY

Note: Sequence data for BJY chickens have been submitted to GenBank with accession numbers EU747285 to EU747296.

a Locus (or Base) located within exon 2.

Of the total 55 SNPs in MHC B-F exon 2 of both breeds, 49 were non-synonymous; 38 in WL and 42 in BJY. Overall, there were 19 amino acid changes that differed between the WL and BJY.

### Association of single nucleotide polymorphisms (SNPs) in the major histocompatibility complex (MHC) B-F exon 2 with immune traits in two breeds

#### White Leghorns (WLs)

Excluding the synonymous mutations and partial SNPs that lacked a sufficient statistical basis, ten loci were analyzed for association of SNP haplotypes and immune traits in WL (Table IV). Of these, six SNPs at loci 69, 218, 220, 221, 223, and 235 were associated with ND titers, three SNPs at 214, 217, and 232 were associated only with AI titers, and one SNP at locus 182 was related to SRBC titers.

**Table IV:** Effects of the SNP genotypes on antibody titer of AI, ND, and SRBC in the White Leghorn chickens.

			Immune trait (log_2_)		
			
SNP position	Alleles (number of birds)	Amino acid change	AI	ND	SRBC
69	G/G (147)	T/A	8.10±1.05	9.62±0.77^a^	8.59±1.36
	A/G (35)		7.83±1.08	9.13±1.06^b^	9.19±1.28
182	A/A (70)	G/R G/S	8.03±1.14	9.52±0.87	8.92±1.43^a^
	A/T (64)		8.14±0.92	9.60±0.82	8.31±1.11^b^
214	T/T (50)	I/S D/V	7.82±1.20^b^	9.43±0.88	8.81±1.51
217	A/A (50)				
	T/G (84)		8.20±0.95^a^	9.63±0.81	8.61±1.27
	A/T (84)				
218	C/C (27)	D/N D/V	7.84±1.00	9.1±0.93^B^	9.10±1.18
220	G/G (27)	R/S R/N R/K			
221	C/C (27)	R/S R/N R/K			
223	A/A (27)	E/V D/V			
235	T/T (27)	I/T			
	C/G (107)		8.11±1.08	9.6±0.80^A^	8.57±1.40
	G/A (107)				
	C/A (107)				
	A/T (107)				
	T/C (107)				
232	A/A (18)	G/D N/S	8.32±1.08^a^	9.81±0.83	9.14±1.29
	A/G (96)		8.06±1.09^a^	9.60±0.78	8.48±1.37
	G/G (20)		7.83±1.08^b^	9.13±1.06	9.19±1.28

Note: The data are expressed as mean±SD. Within a column, values followed by different uppercase letters are significantly different (*P* < 0.01) and values followed by different lowercase letters are significantly different (*P* < 0.05).

Of the six SNPs related to the response to NDV, those at 218, 220, 221, 223, and 235 were linked. Among these six SNPs in seven haplotypes, only one was homozygous. The SNPs at loci 214 and 217, having significant association with response to AI were linked; means for homozygous chickens were distinctly lower than those for heterozygous birds (*P* < 0.05). SNPs at locus 232, also associated with antibody production against AI (*P* < 0.05), with one homozygous type A/A(232) being higher (*P* < 0.05) than the others (A/G(232) and G/G(232)). There were significant effects (*P* < 0.05) of the locus 182 SNP on antibody response to SRBC; mean responses of homozygous individuals were distinctly higher than in heterozygous chickens (*P* < 0.05).

#### Beijing-You (BJY) chickens

All loci were analyzed for their possible association with immune traits and four loci were found to be of interest (Table V). All four SNP locations had effects on the antibody response to NDV, and the SNP at locus 221 was additionally related to the response to SRBC.

**Table V:** Effects of the SNP genotypes on antibody titer of AI, ND, and SRBC in the Beijing-You chickens.

			Immune trait (log_2_)		
			
SNP position	Alleles (number of birds)	Amino acid change	AI	ND	SRBC
158	C/C (3)	K/N	8.33±1.45	9.08±0.83^b^	7.66±0.57
	C/G (52)		8.24±1.37	9.81±0.65^a^	8.23±1.66
	G/G (76)		8.35±1.39	9.97±0.61^a^	8.29±1.39
221	C/C (29)	R/S R/N	8.31±1.13	9.70±0.65^b^	7.79±1.45^B^
	C/A (77)	R/K	8.30±1.22	9.97±0.61^a^	8.42±1.40^A^
192	A/A (68)	I/L I/M	8.28±1.64	9.69±0.82^b^	8.41±1.32
	A/C (44)		8.30±0.99	10.0±0.46^a^	8.04±1.46
194	G/G (33)	I/L I/M	8.38±1.26	10.02±0.57^a^	8.33±1.21
	C/G (53)		8.22±1.24	9.83±0.66^b^	8.35±1.38
	C/C (28)		8.38±1.32	9.81±0.63^b^	7.96±1.59

Note: The data are expressed as mean±SD. Within a column, values followed by different uppercase letters are significantly different (*P* < 0.01) and values followed by different lowercase letters are significantly different (*P* < 0.05).

## Discussion

### Analysis of sequence polymorphisms for exon 2 of the major histocompatibility complex (MHC) B-F gene and immune performance

In the current study, immune performance and polymorphisms in the MHC Class I α1 domain were compared in two distinct breeds. The results indicated that there were significant differences between the two breeds with respect to both immune responses and polymorphisms at the level of both the DNA and proteins. Polymorphism of α1 domain was higher in the BJY than in the WL, both at the nucleotide level. Changes in the structure of the MHC Class I receptor influence its ability to bind and to present peptides to the immune system cells (Lavi et al., 2005). The higher polymorphism in the BJY relative to the WL probably allows the BJY chicks to present a wider variety of peptides on the MHC Class I receptor and perhaps to induce a better response against foreign pathogens. Similar correlation between the level of polymorphism and immune responsiveness against antigens has been demonstrated in some other research (Liu et al., 2002; Ewald & Livant, 2004; Lima-Rosa et al., 2004).

### Association between immune traits and single nucleotide polymorphisms (SNPs) on exon 2 of the major histocompatibility complex (MHC) B-F gene

Chickens are affected by a large variety of pathogens, and evaluation of antibody titer has allowed determining individual genetic differences in antibody reaction kinetics among chicken lines (van der Zijpp et al., 1983; Kreukniet & van der Zijpp, 1990). SRBC, a T-cell dependent antigen requiring relatively greater cooperation of T cells to produce antibodies (Munns & Lamont, 1991), is a commonly used polyvalent antigen for stimulating a humoral immune response, which in birds is considered to reflect a generalized ability to produce antibodies (Gross et al., 1980). AI and ND are the main infectious diseases causing huge losses in chickens and the antibody response to vaccination with AI, ND may indicate the specific ability to mount responses to antigens of major infectious vectors. The main goal of this study was to find SNPs, sufficiently associated with the immune response, that might be studied in the future to improve specific immune reactivity. The results showed that three SNPs were associated with responses to AIV and six were associated with NDV in WL; four SNPs were found with effects on antibody response to NDV in BJY and there were two SNPs associated with antibody responses to SRBC both in WL and BJY. These findings corroborate previous work with other disease vectors (Dunnington et al., 1992; Lakshmanan et al., 1997; Lamont, 1998a,b,c; Karaca et al., 1999; Weigend et al., 2001) and we conclude that the α1 domains of Class I genes may serve as candidate regions to further investigate the genetic control of the humoral immune response in chickens.

The structural basis for peptide binding to mammalian MHC Class I molecules is now fairly well understood (Matsumura et al., 1992; Wilson & Fremont, 1993; Stern & Wiley, 1994; Madden, 1995). Structural analyses have revealed that the side chains of particular amino acids (along with secondary anchors) interact with polymorphic residues that define the binding specificities of a series of pockets (B through F) within the peptide-binding groove. Koch et al. (2007) have reported two structures of the MHC Class I molecule BF2*2101 from the avian B21 haplotype and demonstrated that the binding groove has an unusually large central cavity, which confers substantial conformational flexibility to the crucial residue Arg9 (corresponding to AA61 in the present study), allowing remodeling of key peptide-binding sites. The coupled variation of anchor residues from the peptide, utilizing a charge-transfer system unprecedented in MHC molecules, allows peptides with conspicuously different sequences to be bound. In our study, a clear relationship between antibody production and variation within exon 2 encoding the α1 domain of the antigen binding pocket of Class I MHC gene was found, but the SNPs identified were not the same for the three antigens and there are different SNPs in different breeds associated with the same antigen. These particular SNPs, may underlie the differences found in immunocompetence between different viral antigens and breeds and explain the resistance of some haplotypes to specific disease. Additionally, these polymorphisms may be useful in further understanding the relatively indiscriminate binding through which MHC Class I molecules present peptides to the immune system.

## Conclusions

In conclusion, this study has clearly shown that genomic variation in exon 2 of the MHC B-F gene in chickens had significant effects on antibody responses to vaccination against SRBC, AIV, and NDV. Different SNPs were shown to be associated with responses to AIV, NDV, and SRBC and some linkage was demonstrated between nucleotide loci. The SNPs detected here, which differed between WL and BJY, have the potential for changing the antigen binding pocket in MHC Class I molecules, possibly accounting for differences in immune re-activity. It is noteworthy that SNPs 180 and 182 generate the non-synonymous mutations Arg, ser, or gly for AA61, the critical amino acid emphasized by Koch et al. (2007). The a/t SNP at 182 was relatively frequent and in WL was significantly associated with antibody response to SRBC. Additional work is needed, with immune traits for other important disease vectors, to confirm and extend the array of polymorphisms within the chicken gene encoding Class I MHC. Such investigation may provide the rationale for future application of Marker-Assisted Selection (MAS) for enhancing immunocompetence in chickens.
